# Influence of Polymeric and Natural Stabilizers on the Green Synthesis of Platinum and Palladium Nanoparticles

**DOI:** 10.3390/nano16130804

**Published:** 2026-06-30

**Authors:** Wiktoria Stachowicz, Klaudia Kunicka, Martyna Rzelewska-Piekut, Magdalena Regel-Rosocka

**Affiliations:** Institute of Chemical Technology and Engineering, Faculty of Chemical Technology, Poznan University of Technology, Berdychowo 4, 61-131 Poznan, Poland; kkunicka4@gmail.com (K.K.); martyna.rzelewska-piekut@put.poznan.pl (M.R.-P.)

**Keywords:** platinum group metals, nanoparticles, green synthesis, stabilizers, surfactants

## Abstract

Platinum and palladium nanoparticles (Pt- and Pd-NPs) were synthesized using a green reduction approach with ascorbic acid (AA) or saponin from Quillaja bark (Qb) as reducing agents and stabilized with conventional polymers (PVP, polyvinylpyrrolidone, PEG, polyethylene glycol) or natural surfactants (CG (coco glucoside), Qb). The influence of stabilizer type on reduction efficiency, particle size, and colloidal homogeneity was investigated. Pt-NPs exhibited consistently high reduction efficiencies (>87%) in all systems, whereas Pd-NPs showed lower efficiencies and greater sensitivity to synthesis conditions. AFM and DLS analyses confirmed the formation of particles within the nanometric range. In AA-based systems, Pt-NPs were generally smaller than Pd-NPs, while the opposite trend was observed in Qb-based systems. Natural surfactants provided effective NP stabilization, low values of polydispersity index (PdI), good size control, and stable nanostructures. The results demonstrated that biosurfactant-based stabilizers, particularly CG and Qb, can successfully replace synthetic polymeric stabilizers in the green synthesis of noble metal NPs, supporting the development of more sustainable and environmentally friendly synthesis approaches.

## 1. Introduction

Platinum group metals (PGMs), such as Pt, Pd, and Rh, are the key elements in modern catalytic technologies due to their unique chemical properties. Their high catalytic activity, chemical stability, and ability to perform complex redox reactions have led to their applications in numerous industrial processes, including environmental protection. Platinum group metal nanoparticles (PGM-NPs) are very promising materials, possessing high catalytic activity and better selectivity than non-precious metal NPs [1,2,3].

The growing interest in nanoscale materials, including metal NPs, stems from their exceptional physical and chemical properties compared to larger-sized materials. These unique properties (large surface area and greater chemical reactivity, magnetic and catalytic properties) have contributed to the development of innovative solutions in many fields of science, including chemical catalysis, medicine, and pharmacy, as well as agriculture, electronics, and the food industry [4,5,6,7]. NPs are most often obtained using physical (e.g., laser ablation, electrical irradiation, mechanical milling), chemical (e.g., chemical reduction, microemulsion techniques, electrochemical processes, hydrothermal method, solvothermal method), and biological methods [8,9,10].

**Figure 1:**
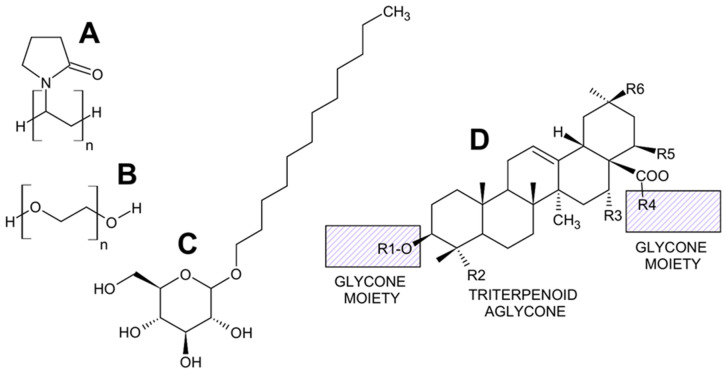
Chemical structures of (**A**) PVP, (**B**) PEG, (**C**) CG, and **(D**) a representative triterpenoid saponin containing glycone and aglycone moieties ((**D**) based on [20]).

Chemical reduction is a frequently used method for obtaining metal NPs. Commonly used reducing agents are alcohols (e.g., ethanol), borohydrides, sodium borohydride, hydrogen gas, sodium citrate, sugars (e.g., glucose), and hydrazine [8,9,10]. Sodium borohydride is successfully used as a reducing agent for platinum [1,11], palladium [1,12], rhodium [1,13,14,15], gold [16] and silver [16,17,18]. Polyvinylpyrrolidone (PVP) [1,13,14,18] and polyethylene glycol (PEG) [19] are mainly used as stabilizers. Particularly, reducers such as sodium borohydride and hydrazine are considered strong reducers but also negatively affect living organisms. Therefore, focusing on green chemistry rules and targeting sustainable development goals, bioderived chemicals are searched to replace the conventional ones. Chemical structures of the selected stabilizers are presented in Figure 1.

PVP and PEG are polymers mainly used to stabilize precipitated NPs and protect them from aggregation. PVP is a non-toxic polymer; it contains a strongly hydrophilic group—the pyrrolidone moiety and a hydrophobic group—the alkyl group (Figure 1A), and is mainly used as a surface stabilizer, growth modifier, NPs dispersant, a shape-control agent and also as a reducing agent [21,22,23,24,25]. Its stabilizing effect is primarily attributed to steric hindrance and adsorption of the carbonyl groups onto the nanoparticle surface, which prevents aggregation and influences particle growth [26]. PEG is a biocompatible polymer (Figure 1B) widely applied across various fields due to its favorable physicochemical properties, including high solubility in both aqueous and organic media, low toxicity, limited immunogenicity, and cryoprotective capability [19,25,27]. PEG stabilizes nanoparticles through surface coordination and steric repulsion, forming a protective polymer layer around the particles and improving their colloidal stability [28,29].

In contrast to chemically derived PVP and PEG, coco glucoside (CG) and saponins from Quillaja bark (Qb) are natural stabilizers. CG is a non-ionic, 100% biodegradable and environmentally friendly glucose derivative obtained by the condensation of alcohol from coconut with glucose from corn, potato or wheat. The molecule of CG consists of a hydrophobic alkyl residue (from alcohol) and a hydrophilic saccharide structure (from glucose), which are linked through a glycoside bond (Figure 1C) [30,31]. The amphiphilic structure of CG enables adsorption onto particle surfaces, while the hydrated glucose moieties provide steric and hydration stabilization that reduces particle aggregation [32]. The saponins, due to their molecular structure, can be used not only as stabilizers but also as reducers [33]. Saponins from *Quillaja saponaria* are a mixture of various compounds obtained primarily from Quillaja bark. The most important components of this mixture are triterpenoid saponins, consisting of a glycone moiety (the sugar part responsible for reduction) and a triterpenoid backbone (aglycone) responsible for stabilization (Figure 1D). Qb stabilizes NPs primarily through a combination of steric and electrostatic repulsion [34]. CG has primarily been described as a stabilizer of micellar, emulsion, and lipid NP systems [35], whereas reports concerning its application in the stabilization of metallic NPs remain limited. Quillaja saponins are mainly described as natural emulsifiers and stabilizers used in colloidal food systems due to their interfacial properties and health benefits [33,36]. They were also reported as reducing and stabilizing agents in the green synthesis of gold NPs (AuNPs) [37]; however, there is still very limited information about their use in the reduction and stabilization of PGMs.

Although numerous studies have described the green synthesis of Pt-NPs and Pd-NPs, comparative studies on conventional polymeric stabilizers and naturally derived biosurfactants, including ascorbic acid (AA)- and Qb-based systems, remain scarce. In particular, natural stabilizers such as saponins and CG are still underexplored despite the widespread use of PVP and PEG.

Therefore, this preliminary work compares the effects of conventional polymeric stabilizers (PVP and PEG) and natural biosurfactants (CG and Qb) on the green synthesis, formation, and stabilization of Pt-NPs and Pd-NPs. In the first four investigated systems, AA was used as the reducing agent together with four different stabilizers (PVP, PEG, CG, and Qb), enabling a direct comparison of the stabilizing effects under identical reduction conditions. Due to the dual nature of saponins, in the subsequent three systems, AA was replaced by Qb in order to preliminarily evaluate whether Qb could also effectively act as a reducing agent while the stabilization was provided by PVP, PEG, or CG.

## 2. Materials and Methods

### 2.1. Reagents

Platinum(IV) chloride (96%, Sigma-Aldrich, Steinheim, Germany), palladium(II) chloride (99.9%, Sigma-Aldrich, Germany), hydrochloric acid (35–38%, Chempur, Poland), L(+)-ascorbic acid (AA, p.a., Chempur, Piekary Śląskie, Poland), sodium carbonate (p.a., Chempur, Poland), polyvinylpyrrolidone (PVP, MW ≈ 55,000, Sigma-Aldrich, Germany), polyethylene glycol 1000 (PEG, United States Pharmacopeia, North Bethesda, MD, USA), coco glucoside (CG, Escent, France), and saponins from Quillaja bark (Qb, ≥10%, Sigma-Aldrich, Germany).

### 2.2. Synthesis of Nanoparticles

Platinum and palladium precursor solutions were prepared by dissolving PtCl_4_ or PdCl_2_ in 0.1 M HCl. The acidic chloride medium was intended to reproduce conditions relevant to hydrometallurgical leachates, where Pt and Pd are present as chloro-complexes (e.g., [PtCl_6_]^2−^, [PdCl_4_]^2−^). The synthesis procedure involved the addition of selected stabilizers (PVP, PEG, CG or Qb) to the precursor solution under constant stirring, followed by the introduction of AA or Qb as a reducing agent. The pH of the system was adjusted to approximately 7 using 1 M Na_2_CO_3_. All synthesis experiments were conducted using a fixed precursor-to-stabilizer-to-reducer molar ratio of 1:1:1. The reaction components were mixed at a constant volumetric ratio of 5:1:1. This fixed-ratio approach was used to ensure consistent and comparable experimental conditions across all systems.

Samples were collected 1 and 24 h after the completion of the synthesis reaction and subsequently analyzed using Atomic Absorption Spectroscopy (AAS) to evaluate the precipitation efficiency and determine the residual concentration of metal ions remaining in the solution after NP formation. The measurements were performed using a ContrAA 300 spectrometer (Analytik Jena, Jena, Germany) equipped with an air–acetylene flame at characteristic wavelengths of 266 nm for Pt and 244 nm for Pd. Additionally, Dynamic Light Scattering (DLS) analysis was carried out to determine the hydrodynamic size distribution of Pt-NPs and Pd-NPs dispersed in aqueous suspensions using a Zetasizer Nano ZS analyzer (Malvern Instruments Ltd., Malvern, UK) operating based on the Non-Invasive Back Scattering (NIBS) technique. The morphology and size distribution of the synthesized NPs were further characterized by Atomic Force Microscopy (AFM) using a Park NX10 system (Park Systems, Suwon, Republic of Korea).

### 2.3. PGM Removal Efficiency Calculation

The obtained metal concentration data enabled the evaluation of changes in Pt(IV) and Pd(II) concentrations in the solutions over time relative to the initial precursor concentrations, as well as the determination of the metal removal efficiency under different experimental conditions. It is important to consider the possibility that metals coordinated by stabilizers could adsorb on the surface of the precipitated NPs. This could result in the removal of these metals from the solution in unreduced form.

The metal removal efficiency (E) was calculated based on the initial and residual metal concentrations in solution, taking into account the corresponding solution volumes. The efficiency was determined from the difference between the initial amount of metal in the precursor solution and the amount of metal remaining in the solution after 24 h, according to the following equation:

(1) $$E=\frac{C_{p}{\cdot V}_{p}-C\cdot V}{C_{p}{\cdot V}_{p}}\times 100\%$$

C_p_—initial concentration of metal ions in the precursor solution (mg/dm^3^); V_p_—initial volume of the metal precursor solution (dm^3^); C—concentration of metal ions remaining in the solution after the reaction (mg/dm^3^); V—total volume of the reaction mixture after addition of all reagents (dm^3^). The calculated efficiency reflects the removal of the total amount of metal from the supernatant under the assumption that the metal is precipitated as NPs. However, the possibility that a fraction of the removed metal remains in an unreduced form associated with the precipitated NPs represents a limitation of the method applied.

## 3. Results and Discussion

### 3.1. PGM Removal Efficiency

**Figure 2:**
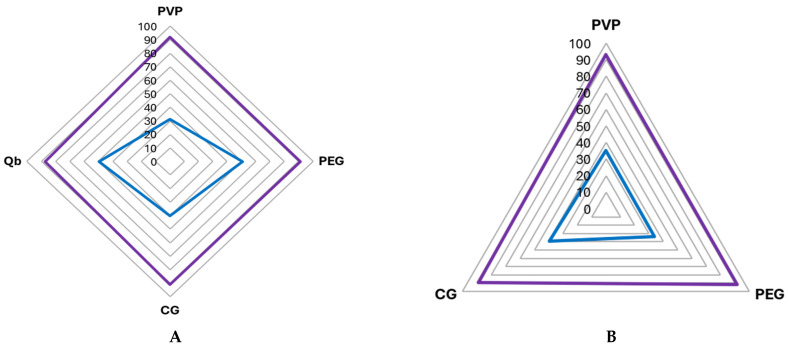
PGM removal efficiencies of Pt-NPs (●) and Pd-NPs (●) synthesized using (**A**) AA and (**B**) Qb as reducing agents and PVP, PEG, CG, and Qb as stabilizers.

In the conducted experiments, Pt-NPs and Pd-NPs were reduced in separate systems, which allowed for an independent evaluation of the influence of the reducing agent and stabilizer on the NP formation reaction. The PGM removal efficiencies obtained for the investigated systems are summarized in Figure 2. All reactions were carried out at a controlled pH of approximately 7, which is favorable for the activity of both AA and saponin Qb [38,39,40].

The results indicate a clear dependence of the PGM removal efficiency on the type of metal NPs formed, while the influence of the reducing and stabilizing agents was less pronounced for Pt than for Pd systems. In all investigated systems, Pt removal efficiency values remained consistently high, ranging from 87.1 to 93.2%, regardless of the applied reducing or stabilizing agent. This suggests that Pt ion reduction proceeded effectively under all tested conditions and that the overall precipitation was relatively insensitive to changes in the stabilizer type. Assuming that the efficiency values predominantly result from Pt-NP precipitation, high efficiencies may additionally be associated with the increase in pH from 1.5 to 7 during the precipitation reaction, which likely promoted Pt precursor hydrolysis and enhanced NP precipitation. The highest Pt removal efficiency was observed for the system containing Qb as the reducing agent and PVP as a stabilizer (93.2%), whereas the lowest value was recorded for the system with AA and stabilized with Qb (87.1%). Nevertheless, the relatively narrow range of efficiencies confirms the high stability and consistency of Pt-NP synthesis across the investigated systems.

In contrast, Pd removal efficiencies were considerably lower and more variable, ranging from 31.2 to 50.9%, indicating a stronger dependence of Pd reduction and stabilization on the composition of the reaction system. For AA-based systems, the highest Pd removal efficiency was obtained in the presence of PEG (50.9%) and Qb (49.6%) as stabilizers, while lower efficiencies were observed for PVP (31.2%) and CG (40.3%). This may suggest that PEG and Qb provided more favorable conditions for Pd nucleation and stabilization, potentially by limiting NP aggregation and improving colloidal stability during synthesis. In the systems where Qb acted as the reducing agent (Figure 2B), Pd removal efficiencies were generally lower than Pt, with values of 35.1, 33.7, and 39.4% for PVP, PEG, and CG, respectively. These results indicate that, although Qb was effective in Pt reduction, its reducing capability toward Pd ions was less efficient compared to AA. The observed differences may also be related to the effect of pH adjustment on Pd species stability and reduction kinetics, as Pd complexes can remain more stable in solution over a broader pH range, limiting the extent of NP precipitation [41,42].

The comparison between Pt and Pd systems demonstrates that Pt ions were reduced substantially more efficiently than Pd ions under analogous experimental conditions. This difference may be associated with the reduction kinetics of Pt and Pd precursors, as well as differences in NP nucleation and growth mechanisms [42]. Overall, the study confirms that both AA and Qb can be successfully applied as reducing agents for PGM NP synthesis.

In the AA + Qb system, it could be difficult to quantitatively separate the reducing and stabilizing contributions of Qb based on the currently available experimental data. Nevertheless, our results suggest that the primary role of Qb in this system is colloidal stabilization rather than reduction. Although Qb may contribute to the reduction process to some extent, the comparative behavior of the investigated systems indicates that its reducing activity is largely suppressed in the presence of AA. This interpretation is supported by preliminary molecular modeling studies, which suggest that AA interacts with the saccharide moieties of Qb. Such interactions may limit the accessibility of the functional groups responsible for the reducing activity of Qb, thereby diminishing its contribution to nanoparticle reduction while preserving its stabilizing effect. Thus, stabilization is considered the dominant function of Qb in the AA + Qb system, while AA acts as the principal reducing agent.

### 3.2. Size of PGM Nanoparticles

**Figure 3:**
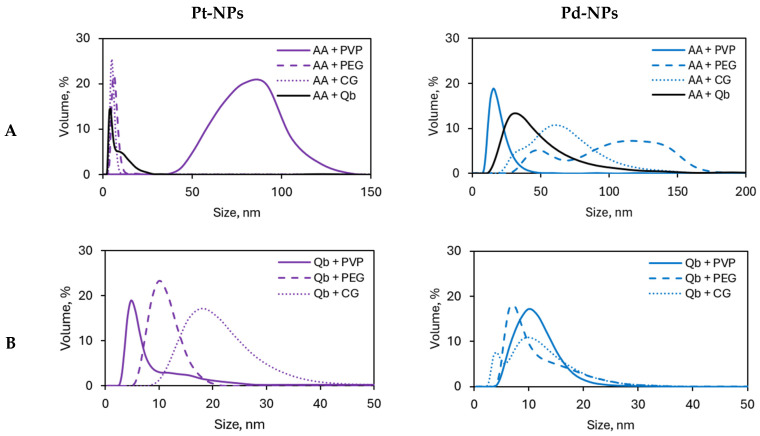
Hydrodynamic size distributions of Pt-NPs (●) and Pd-NPs (●) precipitated with (**A**) AA or (**B**) Qb as a reducing agent, size determined with DLS.

DLS analysis (Figure 3) revealed differences in the hydrodynamic size distributions of Pt-NPs and Pd-NPs, depending on the stabilizing system used. Overall, Pd-NPs exhibited broader size distributions and greater heterogeneity compared to Pt-NPs.

Pt-NPs reduced with AA in the PVP system showed a broad size distribution ranging from approximately 45 to 130 nm with a maximum around 90 nm, suggesting partial NP aggregation. Pt-NPs stabilized with PEG, CG or Qb (Figure 3A) exhibited significantly smaller hydrodynamic diameters (3–30 nm) and relatively narrow distributions, indicating the formation of smaller and more uniform NPs. When Qb was used as a reducer (Figure 3B), relatively small particle sizes below 30 nm were obtained. NPs stabilized with PVP or PEG exhibited narrower and more homogeneous distributions, whereas those stabilized with CG showed a broader distribution shifted toward larger particle sizes, indicating increased aggregation.

Compared with Pt-NPs, Pd-NPs synthesized in the AA-based systems generally exhibited broader and more complex size distributions (Figure 3A). In the AA + PVP system, Pd-NPs showed the narrowest and most uniform distribution, centered around 15–25 nm. In contrast, the AA + PEG system resulted in the broadest distribution, extending beyond 150 nm, indicating pronounced aggregation and high polydispersity. Pd-NPs stabilized with CG also exhibited a relatively broad distribution with particle sizes mainly in the 30–100 nm range. The AA + Qb system showed an asymmetric distribution extending toward larger particle sizes, suggesting the coexistence of smaller NPs and aggregates. When Qb was used as a reducing agent (Figure 3B), all Pd-NP systems exhibited smaller hydrodynamic diameters and more homogeneous size distributions compared with the AA-based systems, indicating the formation of relatively small and stable NPs (<30 nm).

**Figure 4:**
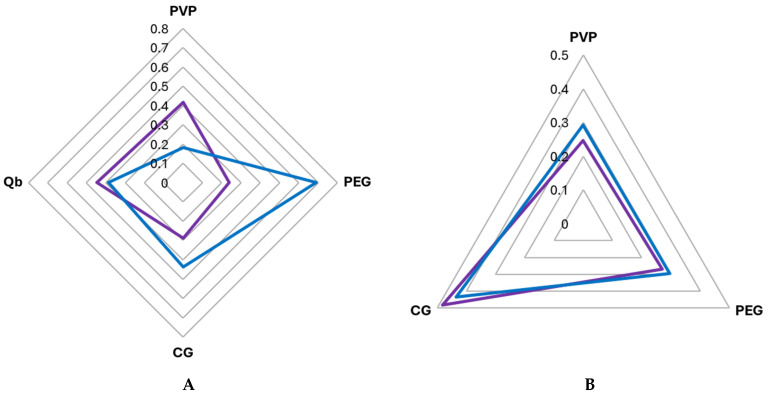
PdI values of synthesized Pt-NPs (●) and Pd-NPs (●) with (**A**) AA or (**B**) Qb as a reducing agent, determined by DLS.

The polydispersity index (PdI) is a dimensionless parameter describing the width of the particle size distribution, ranging from 0 (monodisperse systems) to 1 (highly polydisperse systems), with lower values indicating more uniform systems. In this study, the PdI values of the analyzed systems are presented in Figure 4 confirmed that most analyzed systems were polydisperse. For Pt-NPs, the PdI values in AA + stabilizer systems ranged from 0.241 to 0.446, indicating the influence of the applied stabilizing system on the degree of NPs homogeneity. NPs stabilized with PVP (0.418) and Qb (0.446) showed broader and more heterogeneous size distributions, consistent with the corresponding DLS profiles. In contrast, lower PdI values observed for AA + PEG (0.241) and AA + CG (0.287) systems indicated relatively more homogeneous NP populations. Among the Qb-based systems, NPs stabilized with CG (0.482) exhibited higher polydispersity than for PVP and PEG stabilizers (0.247 and 0.271, respectively) and narrower particle size distributions.

For Pd-NPs, the PdI values generally indicated broader particle size distributions and greater heterogeneity compared to Pt-NPs. Pd-NPs precipitated in the AA + PEG system exhibited the highest PdI value (0.691), consistent with its very broad DLS profile and probable NP aggregation. In the presence of CG and Qb, the PdI showed intermediate values (0.438 and 0.387, respectively), corresponding to moderately broad distributions. Relatively homogeneous Pd-NP populations, indicated by PdI values (0.183, 0.292, and 0.296), were noticed in the AA + PVP, Qb + PVP and Qb + PEG systems. Among the Pd-NPs, the NPs stabilized with CG exhibited the highest heterogeneity within the Qb-based systems, with a PdI value of 0.435, which remained consistent with the observed DLS profile.

### 3.3. Morphology of PGM Nanoparticles

**Figure 5:**
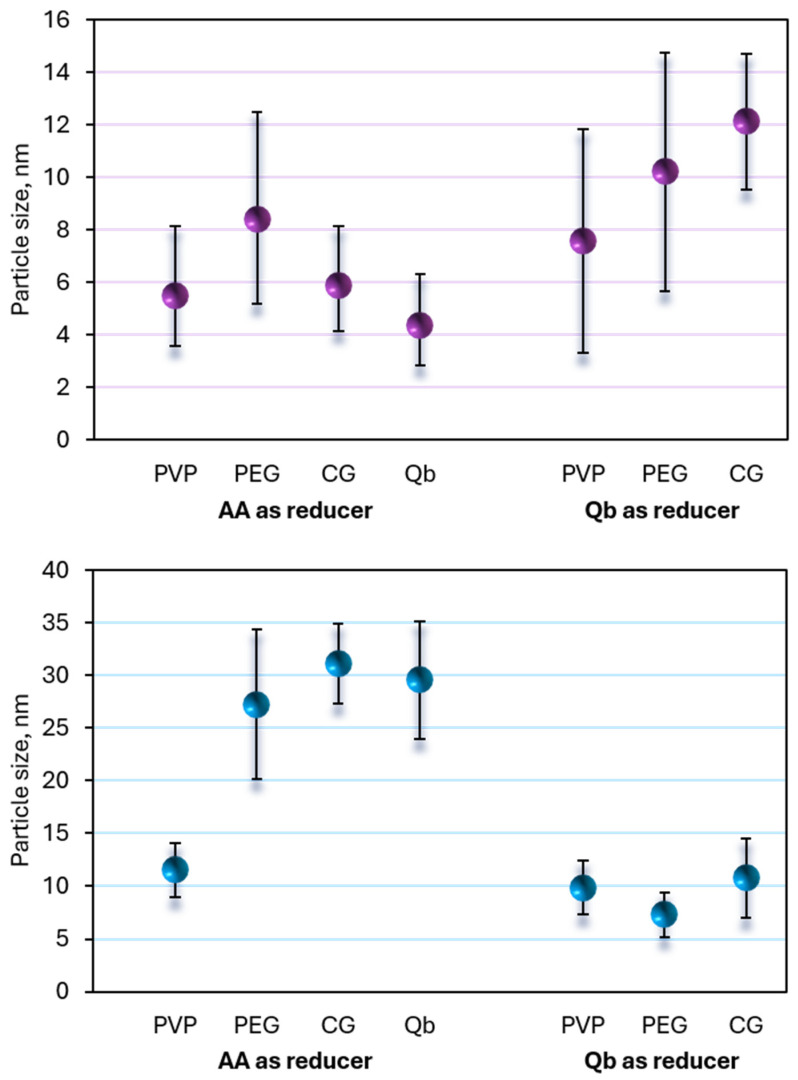
Average particle sizes of Pt-NPs (●) and Pd-NPs (●) obtained from AFM measurements. Error bars indicate the min–max size range.

**Figure 6:**
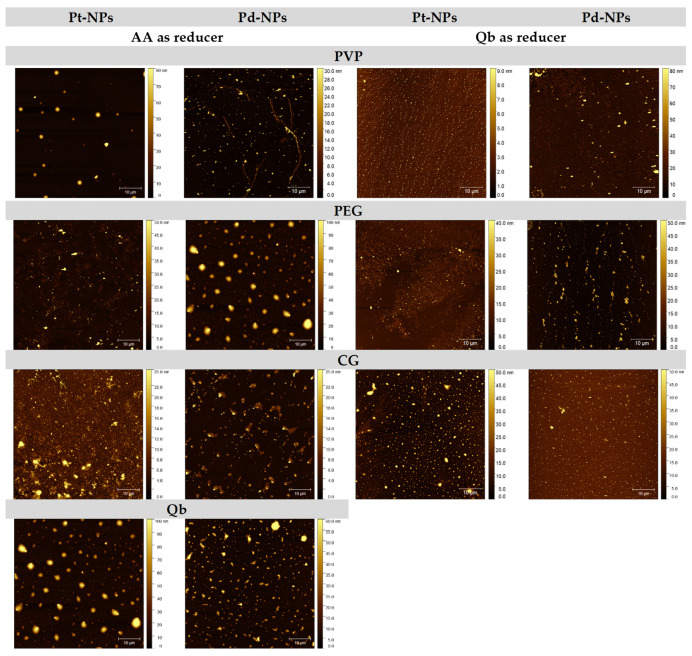
Comparison of AFM images for Pt-NPs and Pd-NPs synthesized under different stabilization and reduction conditions.

AFM analysis revealed differences in the morphology and growth behavior of Pt-NPs and Pd-NPs depending on the applied reducing agent and stabilizer, as shown in Figure 5 and Figure 6.

Pt-NPs exhibited relatively small particle sizes across all investigated systems, with average diameters ranging from 4 to 12 nm. In general, the AA-based systems produced smaller and more homogeneous Pt-NPs, indicating efficient nucleation and controlled growth conditions. The smallest and most uniform Pt-NPs were observed for AA + Qb, suggesting that the presence of Qb effectively limited excessive particle growth and aggregation. In contrast, systems synthesized using Qb as the reducing agent showed an increase in Pt-NP size together with broader particle size distributions. Particularly for PVP and PEG, indicating enhanced particle aggregation and less homogeneous NP growth.

The observed differences in NP size and precipitation efficiency can be attributed to the distinct interaction mechanisms between the stabilizers and the metal precursors. PVP primarily stabilizes nanoparticles through steric hindrance, while its pyrrolidone carbonyl group can coordinate metal ions and metal surfaces, thereby influencing nucleation and growth kinetics [21,43]. PEG likewise provides steric stabilization through the formation of a hydrated polymer layer around particles, but its interaction with metal precursors is generally weaker and less specific than that of coordinating ligands.

In contrast, CG and Quillaja saponin possess amphiphilic molecular structures containing multiple hydroxyl groups together with hydrophobic moieties, enabling strong adsorption at interfaces and efficient reduction in interparticle attraction [44]. Qb saponins are particularly effective because they provide both steric and electrostatic stabilization, thereby suppressing aggregation and flocculation more efficiently than many conventional nonionic polymers. Furthermore, the diverse oxygen-containing functional groups present in these biosurfactants may interact with metal ions through weak coordination and hydrogen-bonding interactions, promoting a more homogeneous distribution of precursor species during precipitation. Similar stabilization mechanisms have been reported for Qb-based NP systems [44].

Compared with Pt-NPs, Pd-NPs exhibited larger particle dimensions and a stronger tendency toward aggregation. The largest Pd-NPs were observed in the AA-based systems with PEG, CG and Qb, where average particle sizes reached 27–31 nm, indicating rapid NP growth and intensified aggregation. Replacing AA with Qb significantly reduced the size of Pd-NPs, with all Qb-based systems exhibiting average particle sizes below 11 nm. These systems also exhibited narrower particle size distributions, indicating improved stabilization and suppression of excessive aggregation.

Thus, good performance of CG and Qb observed in the present study may arise from the combined effects of interfacial adsorption, steric hindrance, electrostatic repulsion, and possible precursor-surfactant interactions, which together limit particle growth and aggregation and enhance precipitation efficiency.

The DLS results were generally consistent with the AFM observations, confirming the formation of Pt-NPs and Pd-NPs within the nanometric range. In most systems, similar trends in particle size evolution were observed for both techniques. However, some discrepancies between AFM and DLS values were noticeable, which is expected due to the different nature of the measurements. DLS determines the hydrodynamic diameter of NPs dispersed in solution, including the solvation layer and possible agglomerates, whereas AFM provides the physical size of individual particles deposited on the surface.

## 4. Conclusions and Future Perspectives

The obtained results clearly demonstrate that both the type of stabilizer and the reducing agent significantly influence the PGM removal efficiency (assuming that it predominantly results from PGM-NP precipitation), particle size distribution, and structural homogeneity of the synthesized Pt-NPs and Pd-NPs. Among the systems stabilized with AA, the highest Pt efficiency was observed for PVP as a stabilizer (92.0%), while the highest Pd efficiency was achieved for PEG (50.9%). In the Qb-stabilized systems, PVP exhibited the best Pt removal efficiency (93.2%), whereas CG showed the highest efficiency for Pd-NPs (39.4%). Overall, Pt removal efficiency remained consistently high in all samples (>87%), while Pd removal was generally less effective and more sensitive to synthesis conditions.

Particle size analysis showed that the relationship between Pt-NP and Pd-NP sizes depended on the stabilizer used. In AA-stabilized systems, Pt-NPs were considerably smaller than Pd-NPs. The smallest Pt-NPs were obtained for Qb (4.37 nm), whereas Pd-NPs in these systems reached larger sizes (11.55–31.11 nm). In contrast, for the Qb-stabilized systems, Pd-NPs were smaller than Pt-NPs, with the smallest Pd particles observed for PEG (7.33 nm), while Pt-NPs ranged from 7.56 to 12.11 nm.

The PdI values demonstrated that NP homogeneity strongly depended on the applied synthesis system. The most uniform distributions were observed for the systems stabilized with PVP or PEG, where comparable and relatively low PdI values were obtained for both Pt-NPs and Pd-NPs, indicating good colloidal homogeneity.

The DLS results were generally consistent with the AFM analysis, confirming the formation of Pt-NPs and Pd-NPs within the nanometric range. Differences between DLS and AFM particle sizes are related to the fact that DLS measures the hydrodynamic diameter of NPs in suspension, including possible agglomerates, whereas AFM determines the physical size of individual particles.

A comparison of polymeric stabilizers (PVP and PEG) with natural surfactant-based stabilizers (CG and Qb) demonstrated that the natural compounds provided stabilization effects comparable to, and in some cases even better than, conventional polymeric agents. The use of natural stabilizers enabled the synthesis of stable Pt-NPs and Pd-NPs with low PdI values and particle sizes remaining within the nanometric range, confirming effective control over NP growth and dispersion. Importantly, the low cost and wide availability of CG, together with the natural origin of Qb, further support their potential as sustainable alternatives to synthetic polymeric stabilizers. These findings indicate that naturally derived surfactants can successfully replace synthetic polymeric stabilizers in the green synthesis of noble metal NPs.

The proposed green synthesis approach proved particularly effective for Pt-NPs, for which high reduction efficiencies were achieved in all investigated systems. In contrast, although Pd-NPs with satisfactory nanometric sizes were also obtained, their reduction efficiency remained noticeably lower, suggesting that Pd-NP synthesis is more sensitive to reaction conditions. Therefore, further optimization of synthesis parameters, especially the concentrations and ratios of reducing and stabilizing agents, may improve Pd reduction efficiency and Pd-NP uniformity.

This work should be regarded as a proof-of-concept study demonstrating the influence of polymeric and natural stabilizers on the green synthesis of Pt-NPs and Pd-NPs. Future research may focus on a systematic expansion of the experimental parameters, including variations in concentrations, ratios, and reaction conditions (e.g., pH, time), in order to further optimize and better understand NP formation and stability. More advanced physicochemical characterization techniques, such as SEM-EDS, TEM, and XPS, could be employed, complemented by kinetic analyses and mechanistic studies to elucidate formation pathways. The system may also be extended toward bimetallic Pt/Pd-NPs to explore potential synergistic effects between the two metals. Ultimately, the approach could be further evaluated under more complex and realistic conditions using genuine hydrometallurgical leachate solutions.

## Data Availability

Data is contained within the article. The raw data of the metal determination is available on request.

## Abbreviations

The following abbreviations are used in this manuscript:

PGMs	Platinum Group Metals
NPs	Nanoparticles
PVP	Polyvinylpyrrolidone
PEG	Polyethylene glycol
CG	Coco glucoside
AA	Ascorbic acid
Qb	Saponin from Quillaja bark
AAS	Atomic Absorption Spectroscopy
DLS	Dynamic Light Scattering
AFM	Atomic Force Microscopy
